# Deconstructing delirium in the post anaesthesia care unit

**DOI:** 10.3389/fnagi.2022.930434

**Published:** 2022-10-04

**Authors:** Antara Banerji, Jamie W. Sleigh, Logan J. Voss, Paul S. Garcia, Amy L. Gaskell

**Affiliations:** ^1^Department of Anaesthesia, Waikato Clinical Campus, University of Auckland, Auckland, New Zealand; ^2^Department of Anaesthesia and Pain Medicine, Waikato District Health Board, Hamilton, New Zealand; ^3^Department of Anesthesiology, Chief Neuroanesthesia Division, Columbia University Medical Center New York Presbyterian Hospital – Irving, Columbia University, New York, NY, United States

**Keywords:** PACU delirium, anesthesia, cognition, attention, disorganized thinking, 3D-CAM

## Abstract

The course of neuro-cognitive recovery following anaesthesia and surgery is distinctive and poorly understood. Our objective was to identify patterns of neuro-cognitive recovery of the domains routinely assessed for delirium diagnosis in the post anaesthesia care unit (PACU) and to compare them to the cognitive recovery patterns observed in other studies; thereby aiding in the identification of pathological (high risk) patterns of recovery in the PACU. We also compared which of the currently available tests (3D-CAM, CAM-ICU, and NuDESC) is the best to use in PACU. This was a *post hoc* secondary analysis of data from the Alpha Max study which involved 200 patients aged over 60 years, scheduled for elective surgery under general anaesthesia lasting more than 2 h. These patients were assessed for delirium at 30 min following arrival in the PACU, if they were adequately arousable (Richmond Agitation Sedation Score ≥ −2). All tests for delirium diagnosis (3D-CAM, CAM-ICU, and NuDESC) and the sub-domains assessed were compared to understand temporal recovery of neurocognitive domains. These data were also analysed to determine the best predictor of PACU delirium. We found the incidence of PACU delirium was 35% (3D-CAM). Individual cognitive domains were affected differently. Few individuals had vigilance deficits (6.5%, *n* = 10 CAM-ICU) or disorganized thinking (19% CAM-ICU, 27.5% 3D-CAM), in contrast attention deficits were common (72%, *n* = 144) and most of these patients (89.5%, *n* = 129) were not sedated (RASS ≥ −2). CAM-ICU (27%) and NuDESC (52.8%) detected fewer cases of PACU delirium compared to 3D-CAM. In conclusion, return of neurocognitive function is a stepwise process; Vigilance and Disorganized Thinking are the earliest cognitive functions to return to baseline and lingering deficits in these domains could indicate an abnormal cognitive recovery. Attention deficits are relatively common at 30 min in the PACU even in individuals who appear to be awake. The 3D CAM is a robust test to check for delirium in the PACU.

## Introduction

The mechanistic details of the patterns and processes of “neurocognitive recovery” after general anaesthesia and surgery are not yet well characterized (Card et al., 2015; Cascella et al., 2020). In a study on healthy young volunteers (without surgery). Mashour and colleagues found that neurocognitive recovery from anaesthesia was a stepwise piecemeal process, in which the parts of the brain that controlled attention and reaction time (tasks assessing working memory/executive function) took longer to recover from isoflurane anaesthesia compared to those parts required for arousal and abstract matching abilities (pre-frontal cortex; Mashour et al., 2021). However, it is unclear whether the same patterns of recovery occur in elderly patients after anaesthesia and surgery; and at what point should delayed recovery in these cognitive domains be considered to have transitioned to a pathological trajectory i.e., Post Anaesthesia Care Unit (PACU) delirium – a harbinger of increased risk of delirium in the ward, long term cognitive decline and increased length of hospital stay (Neufeld et al., 2015; Hesse et al., 2019).

The diagnosis of delirium is based on clinical assessment guided by standard criteria. Whilst the ICD or DSM criteria are widely accepted as “gold-standard” for the diagnosis of delirium, this is not similarly established for emergence or PACU delirium. Additionally, the DSM criteria have changed with time and will continue to do so as studies attempt to characterize the core domains of delirium more rigorously. Of note, “experts” at the diagnosis of delirium will be unfamiliar with the immediate postsurgical patient (especially the effect of residual surgery and anaesthesia drugs on the brain networks and consequently on cognitive recovery). Most tools used for formal delirium assessment are too complex, non-specific and time consuming for use in distressed patients in a busy PACU. Additionally, they conflate different subdomains, and force a binary construct onto a continuum of several parallel cognition recovery streams.

However, there are various established delirium screening tests currently widely used in the PACU, (3D-CAM, CAM-ICU, and NuDESC) which assess the four cognitive domains defined by DSM-5 (an acute change in mental status, inattention, disorganized thinking, an altered level of arousal; European Delirium Association and American Delirium Society, 2014). The most detailed study of cognitive recovery following general anaesthesia was done by Mashour et al. with detailed testing across six cognitive domains. However, this level of detail is impractical in the clinical environment. We needed a more pragmatic and clinically relevant tool to characterize the neurocognitive state in PACU.

To help us understand cognitive recovery in the PACU better, we decided to deconstruct the sub-domains of delirium assessments. This manuscript reports on a sub-analysis of data collected for the Alpha Max study (Gaskell et al., 2019). Our aim was to describe the raw components of the cognitive recovery domains – which are usually combined to make a diagnosis of delirium. In particular, we hypothesized that patients in the clinical environment (after surgery and general anaesthesia) would show distinctive patterns of cognitive recovery conceptually similar to those found in Mashour’s volunteer study. The pre-frontal cortex is believed to play a major role in arousal from anaesthesia (Mashour et al., 2021, 2022; Rokos et al., 2021). By inference, patients that deviated from this pattern might be showing incipient abnormality. We also compared the tests of delirium (3D CAM, CAM-ICU and NuDESC) with one another, to determine which test was most suited to the PACU and evaluation of cognitive recovery patterns.

## Methodology

### Study design and settings

Although a comparison of the different PACU delirium tests was pre-specified, the detailed deconstruction of these tests was a *post hoc* secondary analysis of the dataset collected during the Alpha Max study (Gaskell et al., 2019), a prospective randomized controlled trial using a 2 × 2 factorial design, stratified by pre-operative cognitive score and surgery type. The study included adults aged over 60, scheduled for elective non-cardiac surgery under general anaesthesia that was expected to last at least 2 h. Data were collected over a period from February 2018 to September 2020 at Waikato Hospital, New Zealand. 200 subjects fulfilling the study criteria and completing the study were included for the current analysis. The study was approved by the New Zealand Health and Disability Ethics Committee ref. 17/NTA/56 and had local institutional approval at Waikato Hospital. Australian and New Zealand Clinical Trial Registry, ID:12617001354370, registered on 27/09/2017. Written informed consent was obtained from all patients.

The main aim of the original study was to determine if intra-operative pharmacological manipulations could alter EEG alpha oscillation power, and if that resulted in lower rates of post-operative delirium (Gaskell et al., 2019). The randomized components were: (i) alpha maximization (by titration of desflurane and fentanyl to oscillatory alpha power) vs standard management, and (ii) emergence from a propofol infusion vs standard emergence from volatile anaesthetic. Intra-operative details such as physiological data, total opioids, desflurane and muscle relaxant administered were recorded. In PACU participants were assessed by the research staff 30 min from their time of extubation. The PACU assessment of 3D-CAM and CAM-ICU were performed by three members of the research team; each individual was trained *via* online resources for the screening tests. If they were found to be unresponsive/deeply sedated (Richmond Agitation Sedation Score (RASS) < −2) or in severe pain (a numerical pain scale was used for assessment of pain severity) the researcher would attempt to repeat the assessment after another half an hour (*n* = 16). Individuals who failed to complete 3D-CAM and CAM-ICU assessments due to a low RASS score even at 1 h after extubation (*n* = 8) were graded as having delirium (with all four domains affected). As per the DSM-5, a severely reduced level of arousal above the level of coma should be considered as having severe inattention and thus delirium (European Delirium Association and American Delirium Society, 2014). We acknowledge that sedation is a term associated with a certain degree of ambiguity when used in different settings. The term sedation used throughout this manuscript refers to individuals with a RASS ≤ −3(moderate sedation; brief awakening to voice and eye opening but no eye contact; Sessler et al., 2002). We have considered a score of ≥ −2 as an adequate level of arousal to enable the other cognition assessments to proceed.

Pre-operative data collection included: Baseline standard demographic data; Montreal Cognitive Assessment (MoCA) score. A score of less than 23 was considered an impaired MOCA for the study, MOCA scores were adjusted for level of education (Nasreddine et al., 2005). Short 3D-CAM which included three questions assessing attention: repeating 4 numbers backward, repeating the days of the week backward, and reciting the months of the year backward (MOTYB; Marcantonio et al., 2014; Emrani et al., 2018).

Assessments of delirium in the PACU at 30 min included:

1. 3D-confusion assessment method (3D-CAM) and the 3D-CAM severity score. Information recorded included deconstruction of the features of delirium, along with a severity score for each feature. This feature severity score was calculated based on the CAM-S SF (Vasunilashorn et al., 2016). More details regarding the 3D-CAM and how certain questions were modified for our study is available in the Supplementary Data.
2. CAM-ICU. Special focus was the attention test part of the CAM-ICU, also known as the “Ten-letter vigilance “A” task (Ely et al., 2001)”. Here the patients were given 10 letters (“SAVEHAART”) and required to squeeze the investigators hand each time the letter A was mentioned. Either missing the alphabet or squeezing the finger at a different alphabet was considered as errors. All those with >2 errors were scored as having impaired vigilance. CAM-ICU also includes 4 questions to assess for disorganized thinking. More than one error on these 4 questions was classified as having disorganized thinking.
  We extrapolate our results from tests of disorganized thinking on the CAM-ICU and the 3D-CAM as being conceptually similar – but not identical – to the Abstract Matching test that Basner et al. performed, since both reflect the same neurocognitive domains of higher mental functions (Basner et al., 2015; Mashour et al., 2021).
3. The NuDESC (Nursing Delirium Screening Scale; Gaudreau et al., 2005).
4. RASS (Richmond Agitation Sedation Scale).
5. Speech Language Assessment (Details in Supplementary Data).

Table 1 illustrates all the postoperative assessments performed during the study, delineated according to the different domains of cognition covered by each test. The same cognitive domains (e.g., inattention) are assessed differently by each of the tests in the AlphaMax study.

**TABLE 1:** The various postoperative delirium tests used in our study, divided into the individual cognitive domains, compared to those used in Basner and Mashour’s study.

AlphaMax study		Basner et al. and Mashour et al.		

Cognitive domain assessed	Test	Cognitive domain assessed	Test	Brain regions involved
Acute change in mental status (3D-CAM and CAM-ICU)	Subjective and objective assessment	Not assessed		
Attention (3D-CAM)	Month of the year backward, days of the week backward, digit span	Working memory	Fractal 2-back digit symbol substitution	Dorso-lateral pre-frontal cortex, temporal cortex, motor cortex, cingulate, hippocampus
Vigilance (CAM-ICU)	SAVEHAART	Vigilant attention	Psychomotor vigilance test	Pre-frontal cortex, motor cortex, inferior parietal and some visual cortex
Disorganized thinking (3D CAM)	Year, day of the week, place	Abstraction, concept formation	Abstract matching	Pre-frontal cortex
Disorganized thinking (CAM-ICU)	Does a stone float on water? Are there fish in the sea?	Abstraction, concept formation	Abstract matching	Pre-frontal cortex
Disorganized thinking (NuDESC)	a. Disorientation b. Hallucinations c. Inappropriate communication	Abstraction, concept formation	Abstract matching	Pre-frontal cortex
Level of arousal (3D-CAM)	Objective assessment by researcher	Not assessed		
Level of arousal (CAM-ICU)	RASS			
Level of arousal (NuDESC)	a. Psychomotor Retardation b. Inappropriate behavior			
Not assessed			Motor praxis	Sensorimotor speed
Not assessed			Visual object learning	Spatial learning and memory

The four colors represent the four domains assessed: Acute change in mental status = green; Attention = blue; Disorganized thinking = orange; Level of arousal = yellow.

### Conduct of the 3D-confusion assessment method in the post anaesthesia care unit

The 3D-CAM, a short (3 min) assessment, with reported sensitivity of 95% and specificity of 94% has been widely validated as a tool for diagnosing delirium. In a pilot study, we found it to be more sensitive than CAM-ICU, which is only validated for the ICU population and so doesn’t include any testing that require verbal responses from participant, thus missing interesting and important information. In the absence of any more validated tools, we decided to use 3D-CAM as the primary outcome measure.

It incorporates four cognitive domains (acute change in mental status, attention, disorganized thinking, and level of arousal) required for the diagnosis of delirium as per the DSM-5. These domains of cognition are closely linked to one another, and a hierarchical relationship exists between level of arousal and cognition. A certain level of arousal is necessary prior to the assessment of other cognitive domains, especially attention (European Delirium Association and American Delirium Society, 2014). Arousal corresponds to level of consciousness, whereas attention is the ability for sustained focus and is part of the content of consciousness (Cohen, 2011). Impaired attention in turn can have an impact on performance on other domains like orientation. Because patients had a general anaesthetic, by definition, a change in mental status was universally present in all individuals following anaesthesia.

In our study individuals were administered the 3D CAM assessment after a RASS score of ≥ −2 was achieved. At that time this was considered an adequate level of arousal to assess for attention. We assumed all individuals experienced the same mental status change due to exposure to anaesthesia, so only those who exhibited additional changes (after approximately 30 mins following extubation in the PACU) based on responses to the 3D CAM questionnaire were classified as having an acute change in mental status.

### Data analysis

The four 3D-CAM features (Acute change in mental status, Attention, Disorganized Thinking/Orientation and Level of Arousal) were inspected individually for their incidence and relation to one another. The performance under the individual cognitive domains of the other tests (CAM-ICU and NuDESC) were also analysed for a better understanding of cognitive recovery patterns.

Individuals were divided into two groups using the conventional criteria for delirium in the 3D-CAM system. Statistical analysis was carried out using MATLAB R2021a. Data has been presented as mean and standard deviation for continuous data. Categorical data are presented as numbers or percentages (%). *P* values of less than 0.05 are considered statistically significant. Statistical analyses included *t*-test (unpaired) for normally distributed continuous data, chi square or Fisher’s exact test for categorical data, and McNemar test for paired nominal data. Sensitivity, Specificity, Positive Predictive Value (PPV) and NPV (Negative Predictive Value) were used to compare the various delirium tests.

## Results

### Overall incidence of post anaesthesia care unit delirium

**FIGURE 1:**
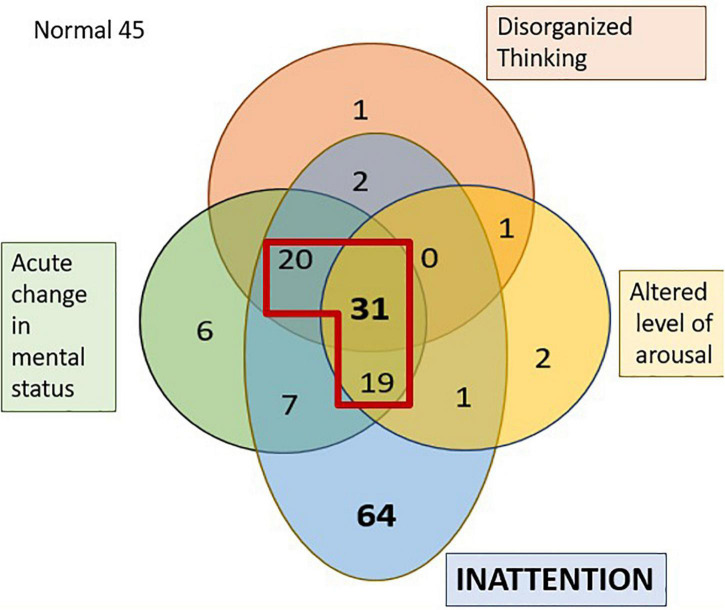
Venn diagram representing the distribution of the four features of delirium in the PACU. The numbers represent the individuals who failed the tests of that specific domain. The section in diagram within the red outline is those diagnosed as having delirium as per the 3D-CAM. 45 individuals had no features of delirium.

The 3D-CAM has the highest reported specificity and sensitivity for detecting delirium; therefore, we used this metric as the primary outcome measure to which we compared other delirium assessments (Jones et al., 2019; Motyl et al., 2020; Aldwikat et al., 2022). The mean time to delirium assessment was 26 min after arrival in PACU. The incidence of PACU delirium was 35% (*n* = 70, red section in Figure 1). Delirium was associated with advanced age, lower BMI, vascular surgery and pre-operative MOCA score, but not surgical duration. Comparison of all other pre-operative variables are available in Supplementary Data. Another 85 individuals had sub-syndromal delirium, that is, one or two features present but not meeting 3D-CAM criteria for delirium (see Supplementary Data).

### Cognitive domains

Figure 1 shows the number of PACU patients who exhibited various combinations of the four features of the 3D-CAM assessment. Certain cognitive domains were more frequently affected than others; almost three-quarters of the patients (72%, *n* = 144) displayed inattention in the PACU (pale blue regions in Figure 1) and a little less than half the patients (41.5%, *n* = 83) exhibited an acute change in mental status (after applying the modification to the 3D CAM used for the Alpha Max study). In contrast, only around a quarter of patients (27.5%, *n* = 55) showed signs of disorganized thinking (red region in Figure 1), but in almost all of these, the disorganized thinking occurred in association with inattention. It was very rare to see isolated problems with disorganized thinking (*n* = 2), altered level of arousal (*n* = 4) or an acute change in mental status (*n* = 6). Note, the 6 patients who were scored as having an isolated acute change in mental status claimed to feel confused, however, observer scoring in the 3D-CAM, NuDESC as well as CAM-ICU did not find them to be categorized as confused. The 3D-CAM algorithm is such that patients reporting subjective confusion only and “passing” the objective confusion questions are still assessed as “positive” for that feature.

The CAM-ICU test established that almost all the patients had an intact level of vigilance (93.5%, *n* = 187) at the time of assessment (based on the vigilance A task). Vigilance is proposed to be the ability to be aware of relevant, unpredictable changes in one’s environment and is a prerequisite for sustained attention (van Schie et al., 2021). Additionally, 81% (*n* = 162) exhibited no signs of disorganized thinking (Disorganized thinking was present in 19% (*n* = 38) CAM-ICU vs 27.5% via the 3D-CAM).

### Attention and sedation

**FIGURE 2:**
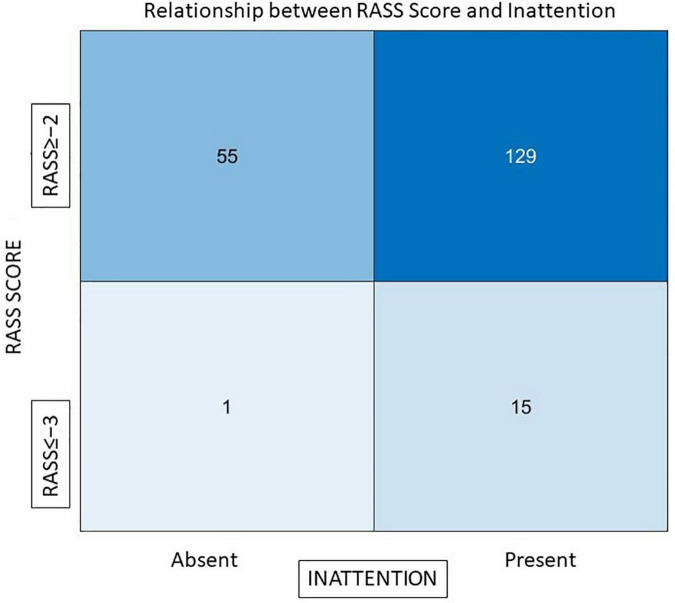
The relationship between RASS scores and inattention. The x-axis represents those who had inattention and those who did not, y-axis represents RASS scores of patients Out of a total of 144 individuals who failed the attention tests 129 (89.5%) had an adequate level of arousal (RASS ≥ −2).

To understand the role of residual sedation in causing inattention, we compared attention tests with RASS scores. There was a statistically significant association between those who were sedated and had inattention (3D-CAM). Even mild sedation caused attention difficulties (out of 31 patients who scored RASS = −2, 29 failed tests of attention), but almost half (*n* = 48/83) of those considered fully awake (RASS = 0) also failed the attention tests, suggesting that a mechanism other than sedative effect of residual drugs has an important role in causing attention problems post operatively. Figure 2 depicts this relationship between sedation (RASS score) and inattention, 89.5% (*n* = 129) of patients who had inattention had a RASS score ≥ −2.

The major cognitive recovery patterns in this study can be summarized as follows.

1. *Arousal and Vigilance*: At 30 min, the level of arousal was adequate in most individuals, RASS ≥ −2 in 91% (*n* = 184), and 93.5% (*n* = 187) had intact vigilance based on CAM-ICU. (Awake enough to be able to follow commands and squeeze fingers when instructed to do so).
2. *Attention* deficits were present at 30 min in 72% (*n* = 144) of individuals. Of the individuals who failed attention tests on the 3D-CAM, 89.5% (129) had an adequate level of arousal (RASS ≥ −2). Of all the individuals with inattention 36.8% (*n* = 53/144) also displayed signs of disorganized thinking.
3. *Disorganized thinking* was more prevalent (27.5%, *n* = 55 3D-CAM;19%, *n* = 38 CAM-ICU) at 30 min in our study. Additionally in our study almost all individuals (53 out of 55) had inattention along with disorganized thinking.

### Comparison of different delirium tests

**FIGURE 3:**
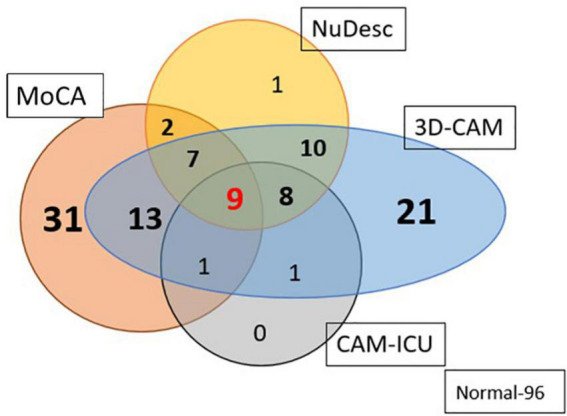
Venn diagram representing the number of patients diagnosed as having PACU delirium based on the three different assessment methods; and their relationship to preoperative cognitive impairment, as detected using the MoCA. 3D-CAM was assumed to be the standard of delirium diagnosis. Out of a total of 70 patients graded as delirium present by the 3D-CAM, the CAM-ICU picked up only 27% and the NuDESC picked out 52.8% (96 individuals had no impairment pre-op and post op).

We compared the various assessments of delirium with one another (Figure 3). Of those classified with pre-operative MoCA impairment (*n* = 63), 47% (*n* = 30) developed delirium in the PACU (chi-square test, *p* = 0.01). The incidence of PACU delirium among those with a normal MoCA (*n* = 137) was 29% (*n* = 40). Individuals with an impaired MoCA had a higher incidence of inattention (*p* < 0.0001, OR = 0.22, CI = 0.09, 0.53) and disorganized thinking (*p* < 0.001, OR = 0.34, CI = 0.17, 0.65). There was a large discrepancy in the numbers of patients diagnosed with delirium based on the 3D-CAM vs the CAM-ICU. A total of 70 patients were graded as having delirium by the 3D-CAM; the CAM-ICU picked up only 19, while the NuDESC was able to identify 37. We feel that individuals with cognitive deficiencies in the PACU are best picked up by the 3D-CAM when compared to NuDESC and CAM-ICU.

## Discussion

Overall, we found a 35% prevalence of delirium in the PACU using the 3D-CAM assessment, similar to results from other studies (Sharma et al., 2005; Neufeld et al., 2013b; Tieges et al., 2018). When separated into individual cognitive domains, inattention was the most prevalent impairment (72%); fewer patients had trouble with disorganized thinking (19%), and only a small proportion of individuals had trouble on tests of vigilance (6.5%). This highlights that the brain recovers from anaesthesia in a piecemeal fashion. Our findings also re-affirm that the challenging nature of the tests of attention used in the 3D-CAM assessment mean that it is probably the ideal test for the PACU, when compared to CAM-ICU and NuDESC.

### Vigilance and disorganized thinking

Multiple studies have found early engagement of the pre-frontal cortex after surgery, as demonstrated by early return of abstraction (Abstract Matching test) and vigilance (Psychomotor Vigilance Test), leading them to believe that the pre-frontal cortex has an important role in arousal from anaesthesia (Huels et al., 2021; Mashour et al., 2021, 2022; Rokos et al., 2021). Our assessment of vigilance and disordered thinking revealed similar results, with majority of individuals performing successfully on the tasks. Vigilance, which was assessed by the CAM-ICU “10-letter Vigilance A task” was intact in 93.5% (*n* = 187) of individuals. Although the tests of disorganized thinking used in the CAM-ICU and the 3D-CAM are relatively simple, they may be extrapolated as mapping onto a fairly similar domain to the Abstract Matching test (Basner et al., 2015). We found no signs of disorganized thinking in most patients in the PACU (CAM-ICU; 81% (*n* = 162); 3D-CAM 72.5% (*n* = 145). The cognitive assessments used for comparison in other studies were more challenging and complex compared to the ones used to derive information in our study. In our situation it was not feasible to subject elderly individuals following 2 h of surgery to an exhaustive battery of complex cognitive tests. Mashour’s study concluded that abstract matching speed and accuracy was relatively normal within 30 min following anesthesia, while in our study a sizable minority (18–20%) still showed signs of disorganized thinking (disoriented/unable to think in an organized fashion). This suggests that signs of disorganized thinking could indicate an abnormal pattern of recovery following anesthesia and surgery.

### Working memory and attention

Working Memory (tested by the Fractal 2 back test and the DSST-Digit Symbol Substitution Test), took up to 2 h to return to baseline in healthy young individuals (Mashour et al., 2021). These are both robust tests of working memory and can be localized to the dorso-lateral pre-frontal cortex as well as certain areas of temporal and motor cortex (Ragland et al., 2002; Usui et al., 2009). This implies that all these areas are required for adequate function of working memory and thereby attention. The importance attributed to inattention in conventional delirium diagnosis needs to be reassessed for the PACU; because the delayed return of attention is probably a component of normal physiological recovery of cognitive domains following anaesthesia – and *not* an indicator of pathological passive delirium.

Inattention is a cardinal feature of delirium diagnosis (Cole et al., 2003; Adamis et al., 2016; Marra et al., 2018; Tieges et al., 2021). Most individuals had *intact* attention at pre-operative assessment, and post operatively most of those who failed the attention tests were *not sedated*. It is believed that tests of attention measure working memory capacity, which includes the ability to control attention (Redick and Engle, 2006; Rudolph et al., 2006). “Working memory refers to the systems that are assumed to be necessary in order to keep things in mind while performing complex tasks such as reasoning, comprehension and learning” (Baddeley, 2010). Although they sound like different entities, both attention and working memory are closely linked. Attention has been further broken down into three subsets, each controlled by a specific network in the brain. These three domains include: (i) an alerting network (frontal and parietal cortices of right hemisphere), (ii) an orienting network for prioritizing sensory input (the superior and inferior parietal lobules, superior colliculus, temporal parietal junction, and frontal eye fields) and (iii) an executive network involved in target detection (i.e., focal attention) and task maintenance (frontal areas, including the anterior cingulate cortex, basal ganglia, and lateral prefrontal cortex; Petersen and Posner, 2012; Chen et al., 2016). The pre-frontal cortex is a key region in the functions of working memory, based on fMRI studies (Christodoulou et al., 2001). It is involved in many aspects of cognition (Braver et al., 1997). Assessments like the “months of the year backward” involve more complex networks from different brain regions (bilateral middle and inferior frontal gyri, the posterior parietal cortex and the left anterior cingulate gyrus) than simpler tasks like vigilance assessments (Meagher et al., 2015).

#### Altered level of arousal and acute change in mental status

An adequate level of arousal is a perquisite to cognitive assessment, however, there is no consensus regarding a clear definition of the same for the PACU patient. Studies vary widely in their time (30 min after extubating or just prior to discharge from PACU) and method of assessment (RASS, Rikers Sedation Score, Aldrete Score; Lepousé et al., 2006; Radtke et al., 2008; Neufeld et al., 2013a; Card et al., 2015; Aldwikat et al., 2022). Our results and observations suggest that a minimum RASS score of −1 and above could be considered an adequate level of arousal since it is sufficient to assess the most complex tasks like attention. An acute change in mental status forms an essential part of conventional delirium diagnosis, but it is universally present in all patients following general anaesthesia which is why its importance in the PACU needs to be reconsidered.

### Comparison of tests of delirium in post anaesthesia care unit

An average adult spends 60–90 mins (Waddle et al., 1998; Panagiotis et al., 2005) in the PACU being constantly monitored with a dedicated nurse overseeing. It is the ideal location to detect those that could benefit from an early intervention, longer PACU stay, extra attention in the wards and most importantly have their pharmacological therapy adjusted keeping in mind a higher risk of delirium (Safavynia et al., 2018).

In our study, CAM-ICU diagnosed only 27% (19 out of 70) of the patients which had been detected as having delirium by the 3D-CAM, possibly because the assessments are much simpler and focus on domains of cognition that return much earlier (Mashour et al., 2021, 2022; Rokos et al., 2021). Hight et al. (2018) found that, due to the step-wise nature of assessment of the CAM-ICU, individuals with intact attention (vigilance) but having disorganized thinking are missed. Sprung et al. concluded that the reason for the low sensitivity of the CAM-ICU was because it was not able to detect mild delirium (Sprung et al., 2017). Similarly, although NuDESC may be a more specific test, it is required to be performed multiple times to detect a change in the level of cognition. Hernandez et al. (2017) also found that neither test was sensitive enough to pick up post-operative delirium. Though NuDESC assesses many domains, it fails to assess attention comprehensively. Since 3D-CAM assess all cognitive domains more exhaustively than the other available tests, we believe it the most suitable tool for assessment of PACU Delirium among the current available tests.

Our article aims to highlight the fact that there are normal as well as abnormal patterns of cognitive recovery after anaesthesia. Slow recovery of attention is probably normal. More research is required into these cognitive recovery patterns following anaesthesia and establishing a timeline of the sequence of return of various cognitive domains would greatly help formulate a more specific tool to detect those at risk of pathological post-operative delirium. We believe that the PACU is the ideal location to identify such individuals. To develop an appropriate tool it is essential to understand the pattern and time course of cognitive recovery, its neuroanatomic basis and its implications. The results we have presented here are the first step toward developing such a tool.

### Limitations

We acknowledge that DSM-5 is the gold standard for diagnosing delirium in the ward. It is a lengthy assessment best performed through periodic assessments over several days. This is not the situation in the PACU, where patients are too ill to be able to tolerate the prolonged assessment required for DSM-5. Currently the role of DSM-5 in this situation is unknown. Our study has based delirium diagnosis on the 3D-CAM, which has been validated in a non-PACU setting. PACU patients are different from those on whom the 3D-CAM was validated; as they are still recovering from the effects of surgery and anaesthesia. Since there is no specifically designed tool available for assessing PACU delirium, we relied on the 3D-CAM.

Our tests for assessing the various cognitive domains were simpler tests and were performed at one single time point in the PACU. But these tests, which are routinely used to screen individuals, are validated across multiple delirium studies, and are also more practical to perform in a clinical setting. The choice of the single evaluation at 30 min was based on the fact that this time point showed maximal separation in recovery between cognitive domains in Mashour’s study, and also it is sufficient time to minimize the effect of slow arousal to confound the results. It is difficult to confidently localize cognitive processes to particular brain regions, primarily because they involve multiple interconnected networks.

## Conclusion

Our study highlights the fact that following anaesthesia and surgery, cognitive domains recover in a piecemeal fashion. Arousal, organized thinking and vigilance are among the earliest cognitive functions to return. So, continued deficits in these domains in the PACU suggest a pathological recovery. Attention is the most frequently affected domain and it is possible that individuals who show deficiencies in this domain are simply recovering “physiologically” from anaesthesia and surgery. Among the tests commonly available to assess delirium in the PACU, it would appear that the 3D-CAM is most suitable, since it incorporates a comprehensive (subjective as well as objective) assessment of all cognitive domains.

## Data availability statement

The original contributions presented in this study are included in the article/Supplementary material, further inquiries can be directed to the corresponding author.

## Ethics statement

The studies involving human participants were reviewed and approved by New Zealand Health and Disability Ethics Committee ref. 17/NTA/56 and had local institutional approval at Waikato Hospital. Australian and New Zealand Clinical Trial Registry, ID:12617001354370, registered on 27/09/2017. The patients/participants provided their written informed consent to participate in this study.

## Author contributions

JS and AG: concept, study design, and initial protocol for the AlphaMax trial. AB: data analysis and manuscript preparation. All authors: manuscript review, revisions and approval of the manuscript.

## Abbreviations

3D-CAM: 3-min confusion assessment method
CAM-ICU: confusion assessment method for the intensive care unit
NuDESC: Nursing Delirium Screening Scale.
