# Supplementary material for: Deconstructing delirium in the post anaesthesia care unit
Source: Front Aging Neurosci. 2022 Oct 4;14:930434. doi: 10.3389/fnagi.2022.930434 (PMC9577324; doi:10.3389/fnagi.2022.930434)
Supplement: Supplementary file 1 [file Data_Sheet_1.PDF]

Registration and eligibility

Participant ID ______________________

Date ______________________

Inclusion criteria

Is the patient 65 years or older? ◯ Yes ◯ No

Is the patient able to provide informed consent? ◯ Yes ◯ No

Is the surgery elective or semi-elective? ◯ Yes ◯ No

Is the surgery non-cardiac? ◯ Yes ◯ No

Does the surgery exclude the head or neck? ◯ Yes ◯ No

Planned volatile based general anaesthesia? ◯ Yes ◯ No

Expected duration of operation > 2 hours? ◯ Yes ◯ No

Exclusion criteria

Does the patient have chronic pain? ◯ Yes ◯ No
(Chronic pain on regular opioids. Codine/panadol etc. are fine)

Is the patient currently using enzyme inducers? ◯ Yes ◯ No
(Carbemazepine/phenytoin/phenobarbital)

Does the patient have documented current alcohol abuse? ◯ Yes ◯ No

Has the participant participated in this study before? ◯ Yes ◯ No

Has the anaesthetist, if known, refused permission? ◯ Yes ◯ No

Result

Eligibile? __________________________________

Consent

Consented?
- ◯ Yes
- ◯ No

Reason for refusal
- ◯ Not eligible
- ◯ Not interested
- ◯ Unavailable for follow-up (e.g. planning to leave the country)
- ◯ Afraid of the risks / too anxious
- ◯ Privacy concerns
- ◯ Not enough time to consider
- ◯ Too busy
- ◯ Other

If other: ________________________________

Date of consent ________________________________

MOCA

Upload photo of completed MOCA
(Hide any patient identifying information)

MOCA score (/30): ________________________________

Neurocognitive impairment? (yes if MOCA is 22 or below)
◯ Yes
◯ No
(If MOCA < 15, do NOT proceed with study)

Short 3D-CAM

DIGIT SPAN

I am going to read some numbers, but I want you to repeat them in backwards order from the way I read them to you. So for example if I said 6-4, you would say 4-6.

[Say digits at rate of one per second]

8-2-4-3

- ☐ 3
- ☐ 4
- ☐ 2
- ☐ 8

Please tell me the days of the week backwards, starting with Saturday.

- ☐ Sat
- ☐ Fri
- ☐ Thurs
- ☐ Wed
- ☐ Tues
- ☐ Mon
- ☐ Sun

Please tell me the months of the year backwards, starting with December

- ☐ Dec
- ☐ Nov
- ☐ Oct
- ☐ Sept
- ☐ Aug
- ☐ Jul
- ☐ Jun
- ☐ May
- ☐ Apr
- ☐ Mar
- ☐ Feb
- ☐ Jan

Backwards questions all correct

- ◯ True
- ◯ False

Pain score

Pain score: have you had any of the following in the last 24 hours? (0 = none of the time; 10 = all of the time)

	0	1	2	3	4	5	6	7	8	9	10
Moderate pain	○	○	○	○	○	○	○	○	○	○	○
Severe pain	○	○	○	○	○	○	○	○	○	○	○
Feeling worried or anxious	○	○	○	○	○	○	○	○	○	○	○

Patient information

Sex
- ◯ Male
- ◯ Female

Age (nearest year): ______________________

Ethnicity
- ◯ NZ Maori
- ◯ Cook Island Maori
- ◯ Fijian
- ◯ Tongan
- ◯ Samoan
- ◯ Other Pacific Island
- ◯ Indian
- ◯ Other Asian
- ◯ Latin American/Hispanic
- ◯ African
- ◯ Middle Eastern
- ◯ NZ European
- ◯ Other European
- ◯ Other

(If patient identifies with multiple ethnicities, choose the ethnicity that is highest in the list)

Height (cm): ______________________

Weight (kg): ______________________

Systolic blood pressure (mmHg): ______________________

Diastolic blood pressure (mmHg): ______________________

Usually wears glasses all the time?
- ◯ Yes
- ◯ No

Wears hearing aids?
- ◯ Yes
- ◯ No

Type of surgery
- ◯ Vascular
- ◯ Orthopaedics/Neurosurgery
- ◯ Urology/General/Gynaecology/Other

Description of operation ______________________

ASA score
- ◯ 1
- ◯ 2
- ◯ 3
- ◯ 4

Patient status at time of operation
- ◯ Able to proceed
- ◯ Unable to proceed, operation cancelled
- ◯ Unable to proceed, MOCA score low
- ◯ Unable to proceed, lost track of patient / no longer contactable
- ◯ Unable to proceed for another reason

Other reason ______________________

Randomisation

Randomisation group

- ◯ Spindle optimisation and wake from propofol
- ◯ Spindle optimisation and wake from volatile
- ◯ Routine care and wake from propofol
- ◯ Routine care and wake from volatile

Surgery, Anaesthetics, and EEG details

Surgery details

Date of surgery __________________________________

Duration of anaesthetic (mins): __________________________________

Duration of surgery (mins): __________________________________

Amount of IV fluid given during surgery (mL): __________________________________

Estimated blood loss during surgery (mL): __________________________________

Total fentanyl (mcg): __________________________________

Propofol during induction (mg): __________________________________

Propofol during emergence (mg): __________________________________

Muscle relaxant used (name): __________________________________

Total amount of muscle relaxant: __________________________________

Catheterised during surgery?
- ◯ Yes
- ◯ No

Epidural during surgery?
- ◯ Yes
- ◯ No

Spinal during surgery?
- ◯ Yes
- ◯ No

Anaesthetists comment

Anaesthetist:
- ◯ Jamie Sleigh
- ◯ Amy Gaskell
- ◯ Anthony Aho
- ◯ John Barnard
- ◯ Rob Carpenter
- ◯ Alan Goodey
- ◯ Madison Goulden
- ◯ Tom O'Rourke
- ◯ Luke Mercer
- ◯ Nico Mostert
- ◯ Arthur Rudman
- ◯ Lucas Sikiotis
- ◯ Henry Wu
- ◯ Other

Other: __________________________________

Pre-specified MAC range __________________________________

Pre-specified ceiling of fentanyl (mcg) __________________________________

How much of a deviation from your usual practice was this protocol?

◯ None
◯ Minor
◯ Major

Comment about ability to keep to the required treatment group:

____

Note any protocol deviations and reasons

____

Photo of anaesthetics chart

EEG details

Entropy data collected?

◯ Yes
◯ No

g.Nautilus cap worn?

◯ Yes
◯ No

Filename of saved EEG and anaesthetics data __________________________

Start time of recording __________________________

Time of extubation __________________________

PACU information

Time at admission to PACU ________________________________

Time to eyes open (mins) ________________________________

Time to PARS / ready for discharge (mins) ________________________________

First recorded temperature post-op (C) ________________________________

Pain score (0-10) ________________________________

Did the patient receive treatment for delirium in PACU?
- ☐ Yes, pharmacological
- ☐ Yes, non-pharmacological (e.g. restraining)
- ☐ No

If yes, please specify: ________________________________

Did the patient receive naloxone?
- ◯ Yes
- ◯ No

Has the patient had vomiting or dry retching?
- ◯ No
- ◯ Once
- ◯ Twice or more

Has the patient experienced nausea?
- ◯ Not at all
- ◯ Sometimes
- ◯ Often
- ◯ All of the time

Did the patient receive an anti-emetic?
- ◯ Yes
- ◯ No

Analgesics given in PACU before cognitive tests

Fentanyl (mcg) ________________________________

Oxynorm (mg) ________________________________

Morphine (mg) ________________________________

Tramadol (mg) ________________________________

Other analgesic (please specify): ________________________________

Total analgesics given in PACU (including those recorded above)

Fentanyl (mcg) ________________________________

Oxynorm (mg) ________________________________

Morphine (mg) ________________________________

Tramadol (mg) ________________________________

Other analgesic (please specify): ________________________________

CAM-ICU

Date of assessment ________________________

Time of assessment ________________________

RASS level of consciousness assessment:

- ◯ 4: Combative (violent, immediate danger to staff)
- ◯ 3: Very agitated (pulls to remove tubes or catheters; aggressive)
- ◯ 2: Agitated (frequent non-purposeful movement, fights ventilator)
- ◯ 1: Restless (anxious, apprehensive, movements not aggressive)
- ◯ 0: Alert and calm (spontaneously pays attention to caregiver)
- ◯ -1: Drowsy (not fully alert, but has sustained awakening to voice, eye opening & contact > 10sec)
- ◯ -2: Light sedation (briefly awakens to voice)
- ◯ -3: Moderate sedation (movement or eye opening to voice, no eye contact)
- ◯ -4: Deep sedation (no response to voice, but movement or eye opening to physical stimulation)
- ◯ -5: Unarousable (no response to voice or physical stimulation)

(If -4 or -5, do not proceed with CAM-ICU)

Has the patient's mental status fluctuated in the past 24 hours?

- ◯ Yes
- ◯ No

(Most likely yes since they have had surgery)

"Squeeze my hand when I say the letter 'A'."

S A V E A H A A R T

- ◯ No errors
- ◯ 1-2 errors
- ◯ 3+ errors

Disorganised thinking

	Correct	Incorrect
Will a stone float on water?	◯	◯
Are there fish in the sea?	◯	◯
Does one kilo weigh more than two?	◯	◯
Can you use a hammer to pound a nail?	◯	◯

Command: "Hold up this many fingers" (hold up 2 fingers)
"Now do the same thing with the other hand" (do not demonstrate)
OR "add one more finger" (if patient unable to move both arms)

- ◯ Correct
- ◯ Incorrect

3D-CAM

Date of assessment ________________________

Start time of assessment ________________________

Is patient able to answer the questionnaire part of 3D-CAM?
◯ Yes
◯ No

If no, why:
◯ Too much pain
◯ Agitated/confused
◯ Unconscious/narcosed
◯ Refused
(Please still complete the observer ratings)

Cognitive function - Orientation

Now I'd like to ask you some questions to check your memory. Don't worry if you don't know the answers.

[You may repeat each question once]

Please tell me the year we are in right now
◯ Correct
◯ Incorrect

If incorrect, what was their response? ________________________

Please tell me the day of the week
◯ Correct
◯ Incorrect

If incorrect, what was their response? ________________________

Please tell me what type of place this is?
◯ Correct
◯ Incorrect
(Hospital)

If incorrect, what was their response? ________________________

Orientation questions all correct
◯ True
◯ False

DIGIT SPAN

Now I am going to read some numbers, but I want you to repeat them in backwards order from the way I read them to you. So for example if I said 6-4, you would say 4-6.

[Say digits at rate of one per second]

7-5-1
☐ 1
☐ 5
☐ 7

8-2-4-3

- [ ] 3
- [ ] 4
- [ ] 2
- [ ] 8

Please tell me the days of the week backwards, starting with Saturday.

- [ ] Sat
- [ ] Fri
- [ ] Thurs
- [ ] Wed
- [ ] Tues
- [ ] Mon
- [ ] Sun

Please tell me the months of the year backwards, starting with December

- [ ] Dec
- [ ] Nov
- [ ] Oct
- [ ] Sept
- [ ] Aug
- [ ] Jul
- [ ] Jun
- [ ] May
- [ ] Apr
- [ ] Mar
- [ ] Feb
- [ ] Jan

Backwards questions all correct

- ◯ True
- ◯ False

PATIENT-REPORTED SYMPTOMS

Now I am going to ask you some questions about how you have been thinking during the past day

[If the respondent answers yes to any of the following questions, probe him/her for more details.]

During the past day have you felt confused?

- ◯ Yes
- ◯ No

(About basic info (i.e. orientation, reason for hospitalisation), not details of medical condition/treatment.)

During the past day did you think that you were not really in the hospital?

- ◯ Yes
- ◯ No

During the past day, did you see things that were not really there?

- ◯ Yes
- ◯ No

((if patient is blind, select N/A and skip))

END OF PATIENT INTERVIEW

OBSERVATIONS

Was the patient sleepy during the interview?

◯ Yes
◯ No
((requires that they actually fall asleep, but is easy to arouse))

Was the patient stuporous or comatose during the interview?

◯ Yes
◯ No
((Difficult to impossible to arouse))

Did the patient show hypervigilance?

◯ Yes
◯ No
((having excessively strong responses to ordinary objects/stimuli in the environment, being inappropriately startled, etc.))

Was the patient's flow of ideas unclear or illogical?

◯ Yes
◯ No
((nonsensical speech, inappropriate answers to questions, contradictory statements or shifting unpredictably from subject to subject))

Was the patient's conversation rambling, inappropriately verbose, or tangential?

◯ Yes
◯ No
((off target responses or telling a story unrelated to the interview))

Was the patient's speech unusually limited or sparse?

◯ Yes
◯ No
((inappropriately brief or stereotyped answers))

Did the patient have trouble keeping track of what was being said during the interview?

◯ Yes
◯ No
((repeatedly asking the interviewer to repeat questions))

Did the patient appear inappropriately distracted by environmental stimuli?

◯ Yes
◯ No
((such as television, people outside the room, roommate's conversations))

Did the patient's level of consciousness fluctuate during the interview?

◯ Yes
◯ No
((frequently falling asleep for part of the interview, but wide awake for part of the interview))

Did the patient's level of attention fluctuate during the interview?

◯ Yes
◯ No
((very inattentive for part of the interview, but attentive for part of the interview -- Note: just getting some questions correct and other incorrect is insufficient to code this feature))

Did the patient's speech/thinking fluctuate during the interview?

◯ Yes
◯ No
((speaks very slowly during part of the interview then very fast, or speech was coherent for part of the interview and then nonsensical))

Comments

____

Time that test finished

____

RESULTS

Feature 1 present: __________________________________

Feature 2 present: __________________________________

Feature 3 present: __________________________________

Feature 4 present: __________________________________

Is the patient experiencing an acute change in their memory or thinking? (compare to pre-op MOCA and short 3D-CAM)

◯ Yes
◯ No

Delirium present? __________________________________

NuDesc

NuDesc is to be completed by the nurse caring for this patient in PACU, as an impression of the whole recovery care episode

Date of assessment __________________________________

Time of assessment __________________________________

	0 (no symptoms)	1 (present but mild)	2 (present and pronounced)
Disorientation (verbal or behavioural manifestation of not being oriented to time or place or misperceiving persons in the environment)	○	○	○
Inappropriate behaviour (e.g. pulling at tubes or dressings, attempting to get out of bed when contraindicated))	○	○	○
Inappropriate communication (e.g. incoherence, non-communicativeness, nonsensical or unintelligent speech)	○	○	○
Illusions/hallucinations (seeing or hearing things that are not there; distortions of visual objects)	○	○	○
Psychomotor retardation (delayed responsiveness, few or no spontaneous actions/words)	○	○	○

Total score: __________________________________

Delirium present? __________________________________

PACU speech-language screen

Date of assessment ________________________

Tell me all the different ANIMALS you can think of.

Animals, 1-15 seconds ________________________

Animals, 16-30 seconds ________________________

Tell me all the different words you can think of that begin with the letter S.

Letter S, 1-15 seconds ________________________

Letter S, 16-30 seconds ________________________

I'm going to say some sentences. I want you to repeat them exactly as I say them. Ready?

The cat chased the bird
○ Correct
○ Incorrect

If incorrect, what did they say: ________________________

They decided to paint the room blue
○ Correct
○ Incorrect

If incorrect, what did they say: ________________________

The local map was small and difficult to read
○ Correct
○ Incorrect

If incorrect, what did they say: ________________________

The boy and girl climbed the hill and admired the view
○ Correct
○ Incorrect

If incorrect, what did they say: ________________________

I'm going to describe something and I want you to tell me its name. For instance, if I said a type of clothing worn on your feet, you would say socks. Do you understand?

A piece of jewellery that tells time ____________________

A large grey animal with a trunk ____________________

A kitchen utensil used to cut bread ____________________

A large instrument with black and white keys ____________________

I want you to tell me about why you're here today. Try to talk for about one minute. Rate the patient's narrative.

	Effortful, no information, incoherent	Occasional pausing, incomplete information, tangential	Fluent, complete information, cohesive
Fluency	○	○	○
Content	○	○	○
Cohesion	○	○	○

QOR-15

Date ______________________

Time ______________________

How have you been feeling in the last 24 hours? (0 = none of the time [poor]; 10 = all of the time [excellent])

	0	1	2	3	4	5	6	7	8	9	10
Able to breathe easily	○	○	○	○	○	○	○	○	○	○	○
Been able to enjoy food	○	○	○	○	○	○	○	○	○	○	○
Feeling rested	○	○	○	○	○	○	○	○	○	○	○
Have had a good sleep	○	○	○	○	○	○	○	○	○	○	○
Able to look after personal toilet and hygiene unaided	○	○	○	○	○	○	○	○	○	○	○
Able to communicate with family or friends	○	○	○	○	○	○	○	○	○	○	○
Getting support from hospital doctors and nurses	○	○	○	○	○	○	○	○	○	○	○
Able to return to work or usual home activities	○	○	○	○	○	○	○	○	○	○	○
Feeling comfortable and in control	○	○	○	○	○	○	○	○	○	○	○
Having a feeling of general well-being	○	○	○	○	○	○	○	○	○	○	○

Have you had any of the following in the last 24 hours? (10 = none of the time; 0 = all of the time)

	10	9	8	7	6	5	4	3	2	1	0
Moderate pain	○	○	○	○	○	○	○	○	○	○	○
Severe pain	○	○	○	○	○	○	○	○	○	○	○
Nausea or vomiting	○	○	○	○	○	○	○	○	○	○	○
Feeling worried or anxious	○	○	○	○	○	○	○	○	○	○	○
Feeling sad or depressed	○	○	○	○	○	○	○	○	○	○	○

Brice Questionnaire

Date of assessment ________________________________

Were you expecting to be completely asleep for this operation?
- ◯ Yes
- ◯ No

What is the last thing you remember before going to sleep? ________________________________

Category:
- ◯ Being in the pre-op area
- ◯ Being with family
- ◯ Feeling the mask on your face
- ◯ Burning or stinging in the IV line
- ◯ Seeing the operating room
- ◯ Hearing voices
- ◯ Smell of gas
- ◯ Other

What is the first thing you remember after waking up? ________________________________

Category:
- ◯ Hearing voices
- ◯ Feeling mask on face
- ◯ Seeing the operating room
- ◯ Being with family
- ◯ Feeling breathing tube
- ◯ Feeling pain
- ◯ Being in the recovery room
- ◯ Being in ICU
- ◯ Nothing
- ◯ Other

Do you remember anything between going to sleep and waking up?
- ◯ Yes
- ◯ No

If yes, what do you remember? ________________________________

Category:
- ☐ Hearing voices
- ☐ Unable to move or breathe
- ☐ Feeling pain
- ☐ Feeling surgery without pain
- ☐ Hearing events of the surgery
- ☐ Anxiety/stress
- ☐ Sensation of breathing tube
- ☐ Other

Did you dream during your procedure?
- ◯ Yes
- ◯ No

If yes, what was your dream about?

____

Were your dreams disturbing to you?
- ◯ Yes
- ◯ No

What was the worst thing about your operation? ________________________________

Category:

- ◯ Anxiety
- ◯ Recovery process
- ◯ Awareness
- ◯ Pain
- ◯ Unable to carry out usual activities
- ◯ Other

Discharge questionnaire

Date of discharge from surgical ward ________________________________

Date of discharge from the hospital where surgery took place ________________________________

Were they transferred to a rehab facility or another hospital?
- ◯ Yes
- ◯ No

Name of hospital or facility ________________________________

Date of discharge from this hospital or facility ________________________________

Length of stay in hospital post-op (days) ________________________________

Final discharge destination
- ◯ Home
- ◯ Independent unit in retirement facility
- ◯ Nursing level care in retirement facility
- ◯ Did not survive to discharge
- ◯ Other

Other: ________________________________

Has the patient had any of the following? (If yes, fill out the serious adverse events form)

	Yes	No
ICU admission	◯	◯
Unplanned HDU admission	◯	◯
Readmission to hospital	◯	◯
Surgical site infection	◯	◯
Falls	◯	◯
Stroke/MI	◯	◯
Death	◯	◯

Comments

____

30 day follow up questionnaire

Date of contact ________________

Days since surgery ________________

Number of days alive and out of hospital 30 days after operation ________________

Patient status
- ◯ Continuing in study
- ◯ Unable to contact
- ◯ Other

Other ________________

Has the patient had any of the following since the last point of contact? (If yes, fill out the serious adverse event form)

	Yes	No
ICU admission	◯	◯
Unplanned HDU admission	◯	◯
Readmission to hospital	◯	◯
Surgical site infection	◯	◯
Falls	◯	◯
Stroke/MI	◯	◯
Death	◯	◯

Comments ________________

12 month follow up questionnaire

Date of contact ____________________

Days since surgery ____________________

Patient status
○ Continuing in study
○ Unable to contact
○ Other

Other ____________________

Has the patient had any of the following since the last point of contact? (If yes, fill out the serious adverse event form)

	Yes	No
ICU admission	○	○
Unplanned HDU admission	○	○
Readmission to hospital	○	○
Surgical site infection	○	○
Falls	○	○
Stroke/MI	○	○
Death	○	○

Comments: ____________________

T-MOCA

Upload photo of completed T-MOCA
(Hide any patient identifying information)

T-MOCA score (/22): ______________________

Serious adverse event form

Date of report ______________________

Start date of the adverse event ______________________

Report related to which serious adverse event (SAE) monitored in this trial:
- ◯ ICU admission
- ◯ Unplanned HDU admission
- ◯ Readmission to hospital
- ◯ Falls
- ◯ Stroke
- ◯ MI
- ◯ Death

Is this SAE related to study intervention
- ◯ No
- ◯ Unlikely
- ◯ Possible
- ◯ Probable

Description of outcome and action taken ______________________

Serious adverse event form 2

Date of report ________________________

Start date of the adverse event ________________________

Report related to which serious adverse event (SAE) monitored in this trial:
- ◯ ICU admission
- ◯ Unplanned HDU admission
- ◯ Readmission to hospital
- ◯ Falls
- ◯ Stroke
- ◯ MI
- ◯ Death

Is this SAE related to study intervention
- ◯ No
- ◯ Unlikely
- ◯ Possible
- ◯ Probable

Description of outcome and action taken ______________________________
