# Supplementary figures and images for: Deconstructing delirium in the post anaesthesia care unit
Source: Front Aging Neurosci. 2022 Oct 4;14:930434. doi: 10.3389/fnagi.2022.930434 (PMC9577324; doi:10.3389/fnagi.2022.930434)

## Confusion Assessment Method for the ICU (CAM-ICU) Flowsheet

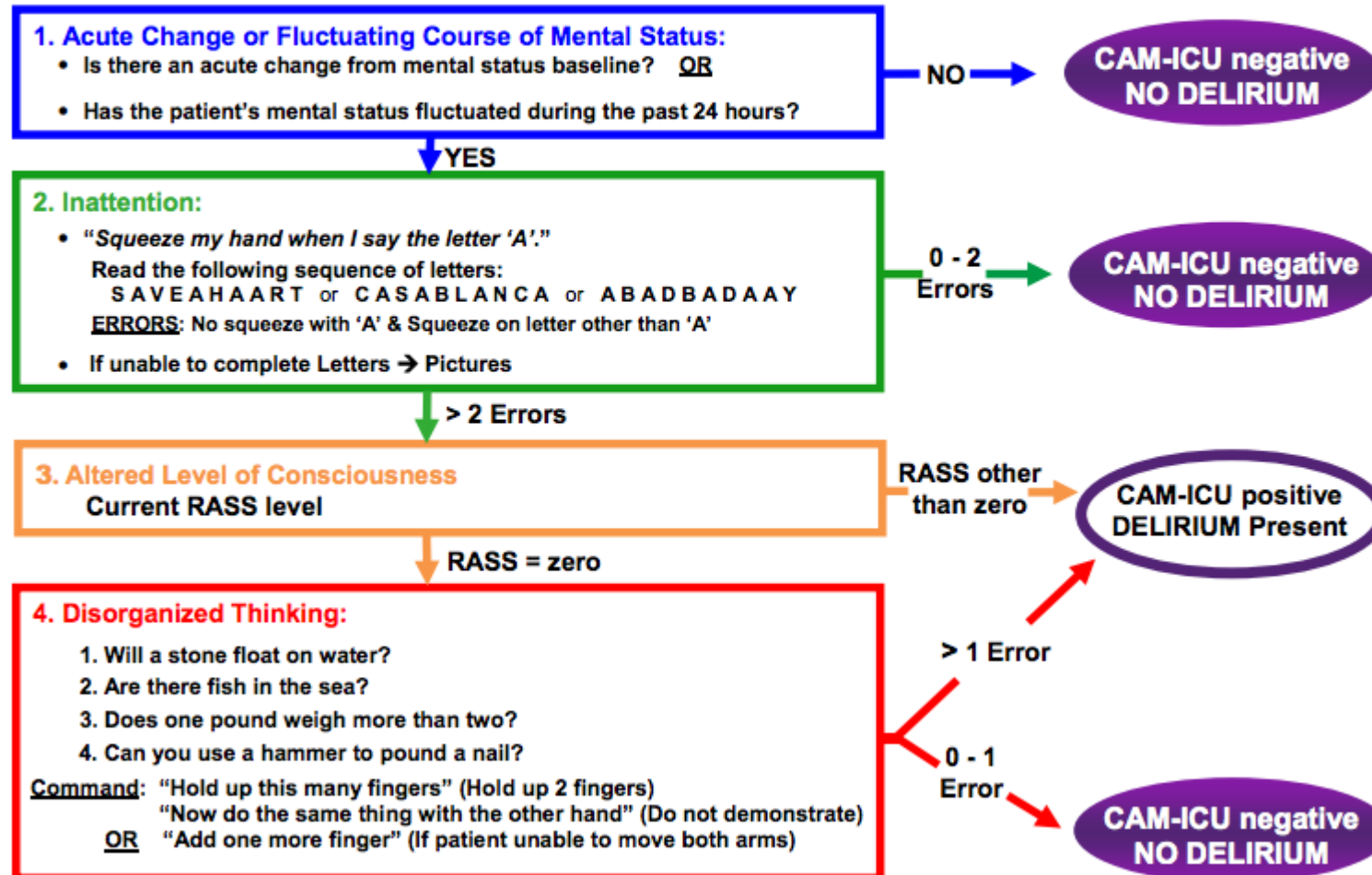

Supplement: Supplementary file 2 [file Data_Sheet_2.PDF]
