# Supplementary material for: Deconstructing delirium in the post anaesthesia care unit
Source: Front Aging Neurosci. 2022 Oct 4;14:930434. doi: 10.3389/fnagi.2022.930434 (PMC9577324; doi:10.3389/fnagi.2022.930434)
Supplement: Supplementary file 3 [file Data_Sheet_3.PDF]

Delirium Assessment Tool

Nursing Delirium Screening Scale

Nu-Desc

Please score the patient at the same time as other assessments in PACU	Score (circle for each element)			
	**0**	**1**	**2**	**SCORE**
**Disorientation** (verbal or behavioural manifestation of not being orientated to time or place or misperceiving persons in the environment)	Alert and orientated to time place and person	Disorientated but easily reoriented	Disorientated on many occasions or not easily reoriented	
**Inappropriate behaviour** (for place and for that person e.g. pulling at tubes / dressings/ climbing out of bed)	Calm and cooperative	Restless but cooperative	Agitated and pulling at devices	
**Inappropriate communication** (for place and person, incoherence, non-communicativeness, nonsensical or unintelligible speech)	Appropriate	Unclear thinking/rambling	Incoherence / nonsensical / unintelligible/ absent	
**Illusions/Hallucinations** Seeing or hearing things that are not there, distortions of visual objects	None	Paranoia, fears	Hallucinations, distortions of visual objects)	
**Psychomotor retardation** Delayed responsiveness, few or no spontaneous words/actions, late response to commands, difficulty rousing patient)	None	Delayed or slow to respond	Excessive sleeping somnolent lethargic	
