# Supplementary material for: Deconstructing delirium in the post anaesthesia care unit
Source: Front Aging Neurosci. 2022 Oct 4;14:930434. doi: 10.3389/fnagi.2022.930434 (PMC9577324; doi:10.3389/fnagi.2022.930434)
Supplement: Supplementary file 4 [file Data_Sheet_4.PDF]

PACU Speech-Language Screen

Patient ID:____________

VERBAL FLUENCY

I'm going to give you a category and ask you to name all the different examples that you can think of from that category in 30 seconds. For instance, if I gave you the category flowers, you might say rose, daisy, etc. Do you understand?

Now go ahead and tell me all the different ANIMALS you can think of.

Record all responses, including repetitions, incorrect exemplars and patient commentary.

**1-15 seconds**	
**16-30 seconds**	

This time I'm going to give you a letter and ask you to name all the different words you can think of in 30 seconds that begin with that letter. For instance, if I gave you the letter "L" you might say life, low, laugh, etc. The only rule is you can't say the names of people or places (so you wouldn't say Lois, Leonard or London). Do you understand?

Now go ahead and tell me all the different words you can think of that begin with the letter S.

Record all responses, including repetitions, incorrect exemplars and patient commentary.

**1-15 seconds**	
**16-30 seconds**	

REPETITION

I'm going to say some sentences. I want you to repeat them exactly as I say them. Ready?

Strike through any word omissions and record any distortions, substitutions or additions.

The cat chased the bird.
They decided to paint the room blue.
The local map was small and difficult to read.
The boy and girl climbed the hill and admired the view.

NAMING

I'm going to describe something and I want you to tell me its name. For instance, if I said a type of clothing worn on your feet, you would say socks. Do you understand?

Record responses.

A piece of jewelry that tells time.	
A large gray animal with a trunk.	
A kitchen utensil used to cut bread.	
A large instrument with black and white keys.	

DISCOURSE

I want you to tell me about why you're here today. Try to talk for about one minute.

Rate the patient's narrative.

	Fluency: 1= effortful, hesitant, no well-formed sentences; 2= occasional pausing, some well-formed sentences; 3= fluent speech with well-formed sentences
	Content: 1= no information; 2= incomplete information; 3= complete information
	Cohesion: 1= incoherent; 2= tangential; 3= cohesive
