# Supplementary material for: Deconstructing delirium in the post anaesthesia care unit
Source: Front Aging Neurosci. 2022 Oct 4;14:930434. doi: 10.3389/fnagi.2022.930434 (PMC9577324; doi:10.3389/fnagi.2022.930434)
Supplement: Supplementary file 5 [file Data_Sheet_5.PDF]

STEP **1**

RICHMOND AGITATION-SEDATION SCALE (RASS)

Level of Consciousness Assessment

Scale	Label	Description
+4	COMBATIVE	Combative, violent, immediate danger to staff
+3	VERY AGITATED	Pulls to remove tubes or catheters; aggressive
+2	AGITATED	Frequent non-purposeful movement, fights ventilator
+1	RESTLESS	Anxious, apprehensive, movements not aggressive
0	ALERT & CALM	Spontaneously pays attention to caregiver
-1	DROWSY	Not fully alert, but has sustained awakening to voice (eye opening & contact >10 sec)
-2	LIGHT SEDATION	Briefly awakens to voice (eyes open & contact <10 sec)
-3	MODERATE SEDATION	Movement or eye opening to voice (no eye contact)

(-1 to -3: VOICE)

If RASS is ≥ -3 proceed to CAM-ICU (Is patient CAM-ICU positive or negative?)

Scale	Label	Description
-4	DEEP SEDATION	No response to voice, but movement or eye opening to physical stimulation
-5	UNAROUSABLE	No response to voice or physical stimulation

(-4 to -5: TOUCH)

If RASS is -4 or -5 → STOP (patient unconscious), **RECHECK later**

Sessler, et al., Am J Repir Crit Care Med 2002, 166: 1338-1344

Ely, et al., JAMA 2003; 286, 2983-2991
