# Supplementary material for: Deconstructing delirium in the post anaesthesia care unit
Source: Front Aging Neurosci. 2022 Oct 4;14:930434. doi: 10.3389/fnagi.2022.930434 (PMC9577324; doi:10.3389/fnagi.2022.930434)
Supplement: Supplementary file 6 [file Data_Sheet_6.PDF]

3D CAM Supplementary data and methodology for assessment:

Pre-op

Three blinded research co-ordinators were responsible for screening, informed consent ,and assessment of the pre-operative MOCA test and shortened 3D-CAM assessment. The order of tests was standardised. The research coordinator who assessed the participant pre-operatively also performed the post-operative assessments in PACU. Online training was completed for both MOCA and 3D-CAM assessments.

Post-op

A standardized method was followed for the post-operative assessments. Thirty minutes after extubation the patients were assessed using the RASS score. If the participant scored a -4 or worse- the assessments were delayed by a further 30minutes. The assessment regime was abandoned if the RASS score was still -4 or worse. If the RASS was -3 or less, then the assessment started with the CAM-ICU followed by the 3D-CAM and finally the speech-language evaluation.

The research coordinator instructed the primary PACU registered nurse to start the NuDesc assessment when the other assessments were performed. NuDesc is a delirium screening tool designed for nurses.

The objective assessment of the 3D-CAM was completed at the end of the assessment timeframe.

Standardization

Before trial recruitment the research team reviewed the assessment tools and set out the standards for meeting the definitions in each tool. This was regularly reviewed throughout the trial recruitment phase. In the case of uncertainty of meeting a specific definition- consensus was sought between the research co-ordinator team. The research co-ordinators remained blinded to the randomised intervention performed in theatre.

1. **Acute Onset or Fluctuating Course**:
   Establishing acute onset and fluctuation of features *requires information beyond what can be elicited on a single bedside assessment,* including information obtained from informants, records on pre-admission functional status and case notes.
   Q: felt confused since waking? Think you were not really in the hospital? See things that were not really there? level of consciousness fluctuates? level of attention fluctuates? Speech or thinking fluctuate?
   AND
2. **Attention**: Preparedness for, and selection of certain environmental or mental stimuli. Reduced ability to direct, focus, sustain and shift attention .
   Q:Digit Span Backwards (3 and 4 numbers),MOTYB, Days of week backwards. Keeping track of interview, inappropriately distracted by environmental stimuli?
   OR

3. **Disorganised Thinking**: Disturbance of organisation and expression of thought. Diverse manifestations including slowing down or speeding up of speech, impaired capacity to make judgements or grasp abstract concepts or loose associations.
   Q:Year, day of the week, Place (orientation). Flow of ideas, conversation rambling, speech limited or sparse.
   OR
   Altered level of consciousness: Global level of behavioural responsiveness and relates to the degree of sensory stimulation required to keep a person awake and attentive. LoA must be sufficient before attention can be formally tested.
   Q:Sleepy during the interview, stuporous or comatose or hypervigilant (subjective assessment).

<u>Modifications to the 3D CAM for the Alpha Max study:</u>

Q21: Additional question if feature 2 was present with either 3 or 4. (Q21: Consult family member or medical record to confirm if patient is showing an acute change in mental status)

5 Step Process for answering Q21 of the 3D-CAM.

1. If feature 4 is present (sleepy/stuporous/hypervigilant) and the patient did NOT display this in the preop recruitment (likely, assessed by comments pre-op) then mark feature 1 as positive.
2. Else, compare orientation questions (Q1-3, feature 3) with MOCA questions about orientation (at the end: year, day, place). If any questions were correct in the MOCA but incorrect in 3D-CAM, then mark feature 1 as positive.
3. Else, compare inattention questions (Q4-7, feature 2) with the short 3D-CAM (same questions) performed pre-op. If TWO or more of the four questions were correct pre-op but incorrect post-op, then mark feature 1 as positive.
4. Else, consider the disorganised thinking observations (Q13-15). If any of these are positive (unclear flow of ideas/rambling/verbose/tangential conversation), and it was NOT present in the pre-op recruitment phase (assessed by comments pre-op), then mark feature 1 as positive.
5. Else feature 1 remains negative.
