# Supplementary material for: Deconstructing delirium in the post anaesthesia care unit
Source: Front Aging Neurosci. 2022 Oct 4;14:930434. doi: 10.3389/fnagi.2022.930434 (PMC9577324; doi:10.3389/fnagi.2022.930434)
Supplement: Supplementary file 7 [file Table_1.PDF]

Additional Supplementary Data:

Table 1: Comparison of cognitive domains assessed by all screening tests used in the study.

**Pre-op**		**Post-op**		
**MOCA**	**3D-CAM (Short)**	**3D-CAM**	**CAM-ICU**	**NuDesc**
		Acute change or fluctuating course in mental state	Acute change or fluctuating course in mental state	
**1. *Attention***		***Inattention***	***Inattention***	
a. Vigilance Task			a. Vigilance Task	
	a.Days of the week backward	a.Days of the week backward		
b. Digits Backward	b.Digits backward	b.Digits Backward		
	c.Months of the year backward(MOTYB)	c.MOTYB		
c.Serial Subtraction				
**2. *Orientation*** (Month/Year/Place/Date/Day)		***Disorganised Thinking*** (Year/Day/Place)+ Subjective assessment	***Disorganised thinking*** (Questions e.g., will a stone float on water)	a.Disorientation b.Illusions/ Hallucinations c. Inappropriate communication
		**Level of arousal**	Altered Level of Consciousness (RAAS)	a.Psychomotor Retardation b.Inappropriate behaviour
3.Abstraction				
4.Delayed Recall				
5.Naming				
6.Visuo-Spatial Orientation				
7.Language				

Table 2: Comparison of demographic data of patients with delirium vs those without delirium.

	**Delirium Present (70)**	**Delirium Absent (130)**	
**Age** (mean)	74.5 (SD± 6.3)	71.6 (SD± 6.8)	***p=0.0041****
**BMI** (mean)	27.8 (SD± 4.8)	29.3 (SD ±5.3)	**p=0.042***
**Surgery** (mean duration)	182.7 (SD ±92.1)	177.0 (SD± 89.8)	p=0.671
**Anaesthesia** (mean duration minutes)	227 (SD ±96.5)	217.7 (SD ±95.8)	p=0.505
**Sex** %,n			
Males	47(67%)	99 (76.1%)	p=0.17
Females	23(33%)	31 (23.84%)	
**Type of surgery**			
Type 1 (Vascular)	33 (47.1%)	39 (30%)	***p=0.0097****
Type 2 (Ortho/Spine)	6 (8.5%)	5 (3.8%)	
Type 3 (Uro, Gyn ,GenSx, others)	31 (44.3%)	86 (66%)	
**ASA Score (American Society of Anaesthesiologists)**			
1	0 (0%)	3 (2.3%)	***p=0.045****
2	21 (30%)	60 (46.1%)	
3	47 (67%)	66 (50.7%)	
4	2 (2.85%)	1 (0.7%)	
**Pre-operative MOCA impairment**	30 (42%)	33 (25%)	p= 0.01

(BMI= Body Mass Index, SD= Standard Deviation), ASA= American Society of Anesthesiology,MoCA= Montreal Cognitive Assessment)

Table 3: Sensitivity and specificity of all tests and sub-tests used in the study:

Test	Sensitivity	Specificity	PPV	PLR	NPV	NLR	Chi-square p-value
**PRE-OP TESTS**							
Pre-op Inattention	14.3%	**90.8%**	45.4%	1.5	66.3%	0.94	
Numbers backwards (pre-op)	42.9%	63.8%	38.9%	1.18	67.5%	0.89	0.35
**MOTYB pre-op**	30.0%	**78.5%**	42.8%	1.39	67.5%	0.89	0.18
MOCA delayed recall	61.4%	53.8%	41.7%	1.33	72.2%	0.71	0.039
MOCA orientation	18.6%	**94.6%**	65.0%	**3.44**	68.3%	0.86	**0.003**
**Pre-op MOCA**	42.9%	**74.6%**	47.6%	1.68	70.8%	0.76	0.01
**POST-OP TESTS**							
Post op Inattention	68.6%	80.8%	65.7%	3.56	82.7%	0.38	
Numbers Backwards (post-op)	91.4%	50.0%	50%	1.82	91.4%	0.17	<0.0001
**MOTYB post-op**	**74.3%**	**73.8%**	60.4%	2.84	84.2%	0.34	**<0.0001**
CAM ICU attention	20%	100%	100%	Inf	71.6%	0.71	<0.0001
**CAM ICU**	72.9%	0.0%	100%	Inf	71.8%	0.72	0.378
**NuDesc**	48.6%	97.7%	91.8%	21.04	77.9%	0.52	<0.0001
